# Risk assessment of land subsidence based on GIS in the Yongqiao area, Suzhou City, China

**DOI:** 10.1038/s41598-024-62108-w

**Published:** 2024-05-18

**Authors:** Longfei Chai, Lu Wei, Pengjie Cai, Jiankui Liu, Jia Kang, Zhen Zhang

**Affiliations:** 1 Anhui Institute of Geo-Environment Monitoring, Hefei, 230001 Anhui China; 2 IMM, State Key Laboratory for Mineral Deposits Research, School of Earth Sciences and Engineering, Nanjing University, Nanjing, 210093 China; 3 School of Geomatics, Anhui University of Science and Technology, Huainan, 232001 China

**Keywords:** Land subsidence, Risk assessment, GIS, Index system, Hydrology, Natural hazards

## Abstract

This study focuses on the Yongqiao District in Suzhou City, Anhui Province, China, aiming to analyze the current situation of ground settlement and its influencing factors in the area. The selected risk indices include settlement rate, cumulative settlement amount, groundwater level drop funnel, thickness of loose sediment layer, thickness of soft soil layer, and the number of groundwater extraction layers. Additionally, vulnerability indices such as population density, building density, road traffic, and functional zoning are considered. An evaluation index system for assessing land Subsidence risk was established. The risk evaluation of land Subsidence was conducted using the Hierarchical analysis-composite index method and ArcGIS spatial analysis, The evaluation results show that the area of higher risk area is about 2.82 km^2^, accounting for 0.96% of the total area, mainly distributed in the area of Jiuli village, Sanba Street. The middle risk area is distributed around the higher area, with an area of about 9.18 km^2^, accounting for 3.13% of the total area. The lower risk areas were distributed in most of the study area, covering an area of 222.24 km^2^, accounting for 75.82% of the total area. The low risk assessment area is mainly distributed in Bianhe Street and part of Zhuxianzhuang Town, with an area of about 58.88 km^2^, accounting for 20.09% of the total area. The findings of this study are not only crucial for informing local policies and practices related to land use planning, infrastructure development, and emergency response but also enhance our understanding of the complexities of land Subsidence processes and their interactions with human activities, informing future research and practice in environmental risk assessment and management.

## Introduction

Land subsidence, a pervasive geological hazard, manifests in over 150 cities across more than 50 nations, emerging as a global environmental challenge jeopardizing human habitation^1–4^. In fact, within China, extensive land subsidence prevails in major cities situated in the deltas of the Yangtze River, Yellow River, and Pearl River, as well as in the Songliao Plain, areas surrounding the Bohai Sea, and the southeast coastal plain^1,5–9^. As China’s economy burgeons, the consequential impact of land subsidence on urban ecological environments and infrastructures is markedly significant^10–12^. Consequently, a meticulous exploration and study of the deleterious effects of land subsidence becomes imperative^13^.

Recent investigations into land subsidence have predominantly concentrated on monitoring^14^, simulations^15^, causes^16^, and the associated hazards and risks^17^. Notably, remote sensing techniques, exemplified by satellite-based interferometric synthetic aperture radar (InSAR), have witnessed escalating utilization for monitoring subsidence across expansive regions^18^. InSAR, proficient in generating high-resolution deformation maps, facilitates the discernment and surveillance of subsidence occurrences^19,20^. Simultaneously, ground-based monitoring methodologies, encompassing GPS surveys, leveling surveys, and geodetic networks, have been instrumental in tracking ground deformation over temporal scales, providing pivotal data for comprehending subsidence patterns^21,22^. The evolution of advanced numerical models, notably employing finite element analysis and finite difference methods, has enabled the simulation of subsidence processes^23^. These models, integrating geological parameters, groundwater extraction dynamics, and soil compaction considerations, serve to predict and comprehend the multifaceted nature of subsidence phenomena^15,24^. Additionally, integrated models amalgamating hydrological, geological, and geotechnical aspects have enriched the understanding of subsidence processes by encapsulating intricate interactions between diverse influencing factors^25^. Identified contributors to land subsidence include excessive groundwater withdrawal, construction activities, and resource extraction such as mining and oil and gas operations^26,27^. These human-induced activities alter geological and hydrological conditions, precipitating ground movement^28,29^. Furthermore, natural processes, like the dissolution of soluble rocks (karst processes) or natural consolidation of sediments, can also contribute to subsidence^30^. The ramifications of subsidence extend beyond infrastructure concerns, encompassing risks to ecosystems through altered water flow patterns, impacting vegetation and wildlife habitats^31^. Coastal regions face an augmented risk of flooding as subsidence lowers the land surface, enabling seawater ingress^32–34^.

While there is a growing awareness of the risks associated with land subsidence, scant attention has been paid to land subsidence indices, including subsidence value, subsidence rate, and cumulative settlement^35–37^. Furthermore, land subsidence risk assessment typically employs various methods such as empirical models, remote sensing techniques, and numerical modeling approaches ^19,38,39^. Empirical models often rely on historical data and statistical analysis to predict subsidence rates based on factors such as groundwater extraction, soil characteristics, and land use ^38^. Remote sensing techniques, including InSAR and global navigation satellite systems (GNSS), offer valuable spatial and temporal data for monitoring ground deformation ^19^. Numerical modeling approaches, such as finite element or finite difference methods, simulate subsurface processes to forecast future subsidence patterns^39^. However, these methods often encounter challenges in systematically integrating multi-criteria decision-making and expert knowledge. The applicability of the Analytic Hierarchy Process (AHP) method in rapid and regional assessments is promising^40,41^. By employing AHP, this study aims to enhance the accuracy and reliability of land subsidence risk assessment by effectively integrating diverse sources of information and expert opinions. The objective of this research is to address the current gap in land subsidence indicators, including subsidence value, subsidence rate, and cumulative settlement. Notably, previous studies on land subsidence in various cities have lacked comprehensive research utilizing AHP ^19,42,43^. Through a comprehensive analysis of factors influencing land subsidence in the Yongqiao area of Suzhou City, considering the geological background of the region, this study aims to comprehensively assess the risk and vulnerability of land subsidence. Leveraging AHP and GIS technology, it provides a scientific basis for urban planning, disaster prevention, and mitigation strategies, ultimately aiming to support the sustainable development of the region^44–46^.

## Study area

**Figure 1:**
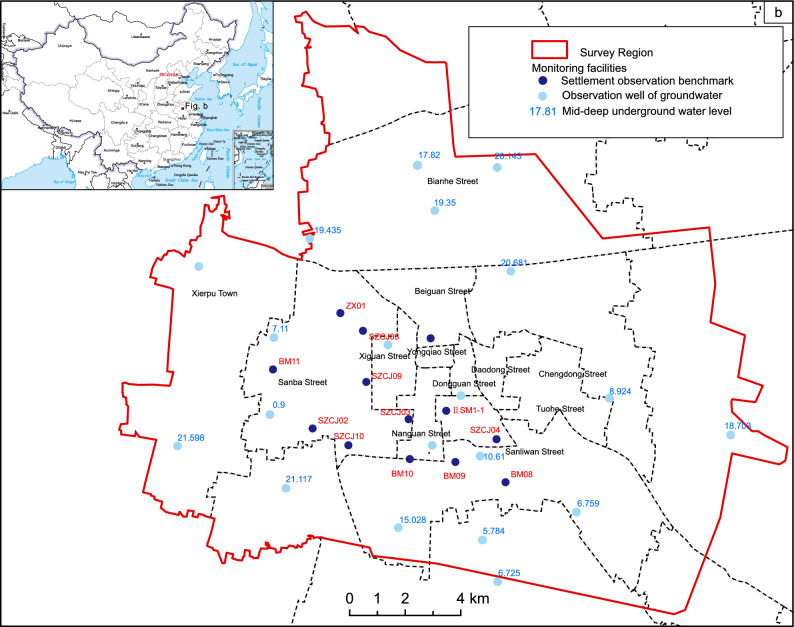
Location of the study area. Arc GIS 10.8 platform (https://www.esri.com) was used for preparing the map for the study area and sample location sites.

**Figure 2:**
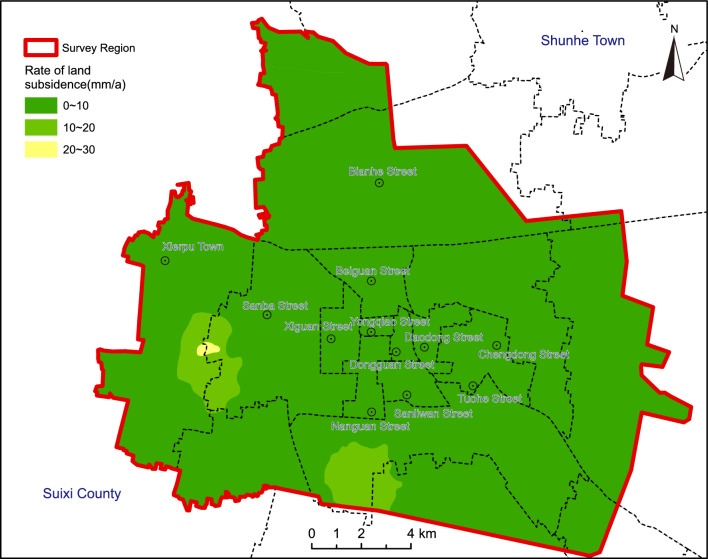
Rate of land subsidence of the Suzhou City. Arc GIS 10.8 platform (https://www.esri.com) was used for preparing the map for the study area.

**Figure 3:**
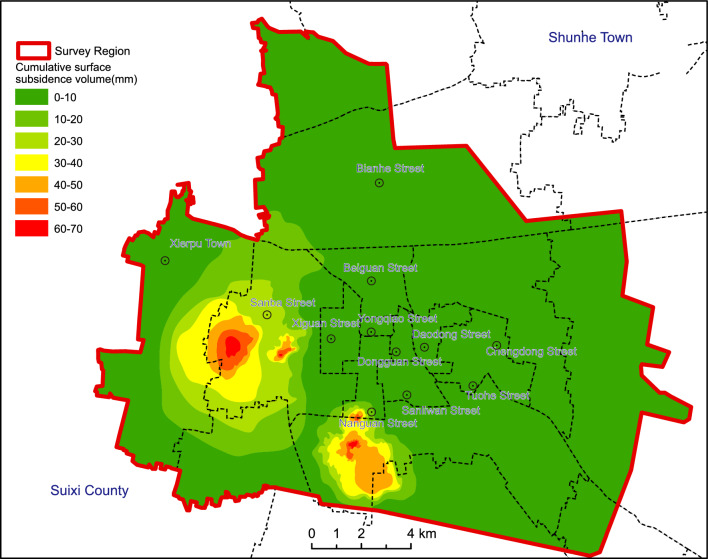
Cumulative surface subsidence volume of the Suzhou City (2019–2022). Arc GIS 10.8 platform (https://www.esri.com) was used for preparing the map for the study area.

The study area encompasses streets and townships in Suzhou City, spanning 294.13 km^2^ (Fig. 1). This region, characterized by high population density and robust transportation networks, experiences an average annual precipitation of 865 mm within a temperate semi-humid climate. The topography is predominantly flat, with minor elevation variations. Geological strata comprise various periods, overlaying a loose Quaternary layer spanning 80–100 m, with thickness variations attributed to alluvial processes, thinner in the north and thicker in the south. Vertical partitioning of the study region delineates shallow, intermediate-deep, and deep aquifers, accounting for aquifer conditions, lithology, hydraulics, and hydrochemistry. The intermediate-deep aquifer, pivotal for groundwater extraction, ranges from 43 to 84 m, comprising 3–4 sand layers, predominantly fine to medium-fine, occasionally coarse sand locally. Well yields exhibit significant variability, resulting in localized cones of depression in areas with concentrated extraction. Historical records document minor land subsidence in Suzhou since the 1990s. A monitoring network established in 2014 covers 57.6 km^2^ around the Xierpu water source, revealing an average subsidence rate of 5–25 mm/year, accumulating to 82 mm from 2014 to 2017. Notably, distinct subsidence regions emerged in Suzhou between 2019 and 2022, particularly around Xierpu and Xiecheng, with rates ranging from 10 to 30 mm/year (Fig. 2). Other areas, such as the Xiqiao water source in Yongqiao and the Economic Development Zone, experienced cumulative subsidence ranging from 10 to 70 mm and 10–60 mm, respectively (Fig. 3). Peripheral zones exhibited subsidence below 10 mm. Land subsidence is attributed to both natural factors, including loose layer thickness and soft soil, and anthropogenic factors, primarily excessive groundwater extraction. The spatial distribution of the cone of depression closely mirrors land subsidence patterns, indicating a direct correlation with groundwater extraction.

## Methods and materials

**Figure 4:**
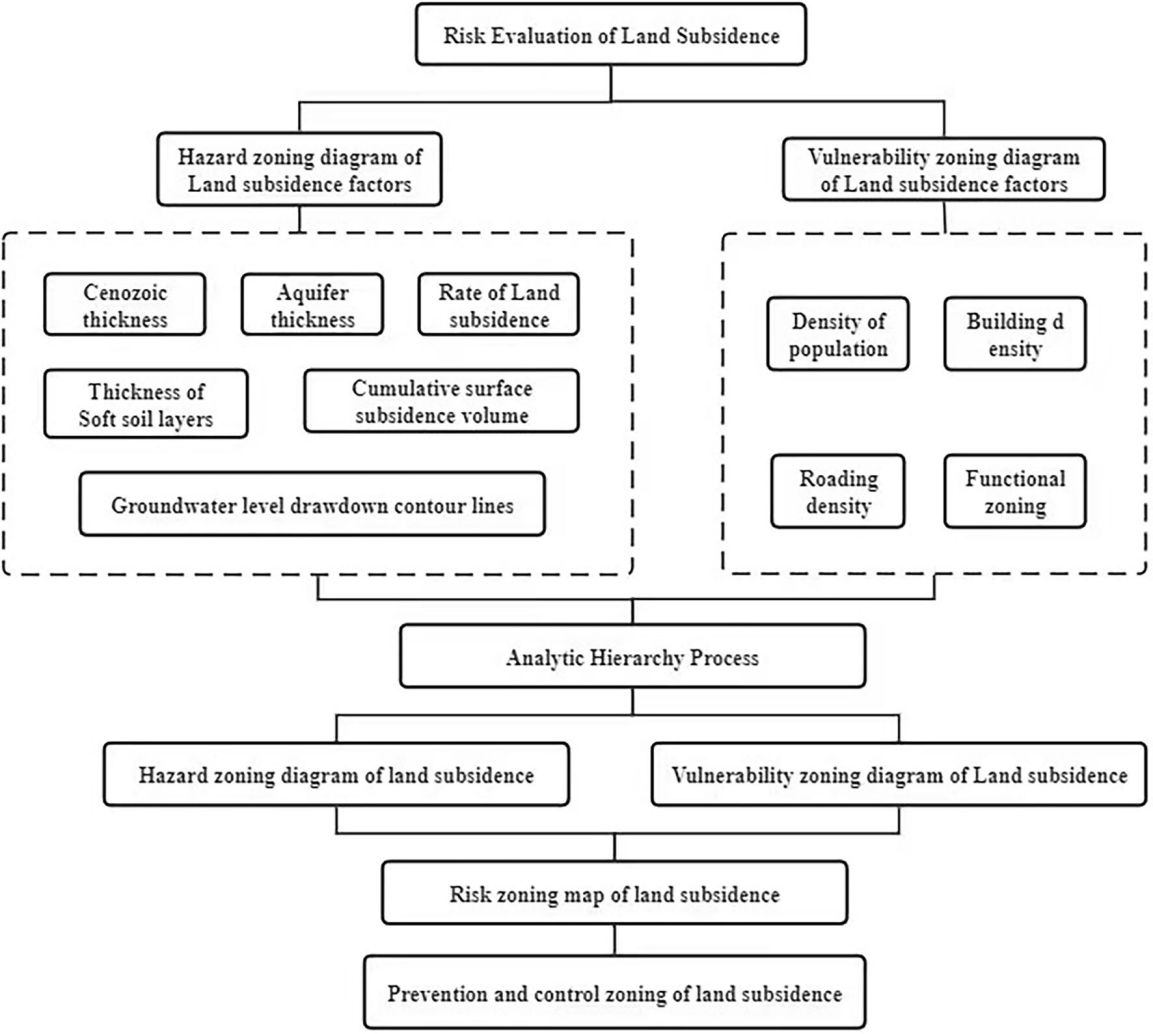
Overall methodological flowchart for this study.

In this study, we address the intricacies of land Subsidence development, considering the complexity of geological conditions, the multitude of contributing factors, and the varying environmental capacities across regions. The overall methodological flowchart is shown in (Fig. 4).

A rational and scientific assessment approach is deemed crucial for effective risk mitigation. We adopt the geological hazard risk assessment model proposed by Dutch scholar Van Westen^47^, represented as Risk = Hazard × Vulnerability, where “Hazard” signifies the likelihood of land Subsidence occurrence (or susceptibility), and “Vulnerability” denotes the consequences of such occurrences. This model is employed to assess the current status of land Subsidence hazards, susceptibility, and vulnerability, leading to a comprehensive risk assessment with recommendations for land Subsidence risk management. ArcGIS spatial analysis capabilities are utilized to conduct a comprehensive evaluation, wherein appropriate factors are selected and the AHP is applied to determine the weights of each factor^48^. Subsequently, the results of various evaluation factors are integrated through analytical procedures using the geological hazard risk assessment model, culminating in the outcomes of the land subsidence risk assessment. The hazard assessment phase, which constitutes a crucial component of the risk evaluation process, entails the evaluation of regional geological hazard probabilities^49^. This assessment combines natural factors, such as geological background and current geological hazard status, with anthropogenic factors including groundwater exploitation and seismic events.

Land subsidence is a complex nonlinear phenomenon, and accurate prediction necessitates the inclusion of causative factors with substantial influence on subsidence occurrence in analysis and prediction models. Considering the subsidence mechanisms and influencing factors discussed earlier, geological background (GB), current land subsidence status (LS), and groundwater exploitation (GE) are identified as primary evaluation indicators for hazard assessment^50^. From a geological background perspective, the Cenozoic strata constitute the primary subsidence layer in the study area, with the thickness of these strata directly impacting subsidence occurrence and development. Generally, thicker Cenozoic strata heighten the risk of land subsidence, emphasizing the importance of their thickness in risk evaluation. Furthermore, soft soil, characterized by high natural water content, substantial porosity, compressibility, low shear strength, and varying physical and mechanical properties among layers, directly correlates with subsidence occurrence. Thicker soft soil layers typically increase the likelihood of land subsidence, making soft soil thickness a critical factor in risk assessment. Land subsidence primarily stems from water pressure loss and aquifer compaction due to excessive groundwater extraction, particularly affecting relatively impermeable clay layers. Consequently, a thicker aquifer and compressible layer correspond to greater land subsidence amplitudes, emphasizing aquifer thickness as a key factor influencing subsidence. In summary, cenozoic thickness, thickness of soft soil layers, and aquifer thickness are designated as secondary indicators. Rate of land subsidence and cumulative surface subsidence volume directly reflect subsidence intensity, thus chosen as secondary evaluation indicators for current land subsidence status. Groundwater exploitation significantly contributes to land subsidence, with areas of intense extraction and central regions of groundwater depression cones correlating with high subsidence rates^51^. The groundwater level drawdown contour line is selected to reflect the extent and intensity of groundwater exploitation. The weights assigned to each indicator are detailed in Table 1.The AHP is employed to establish the hazard assessment model^52,53^, represented by the calculation formula:

**Table1:** Land subsidence hazard evaluation index system and assignment.

Primary index	weight	Secondary index	Index classification	Index weight	Importance assignment
Geological setting	0.2	Cenozoic thickness/m	≤ 50	0.059	1
			50–100		2
			100–150		3
			150–200		4
			≥ 200		5
		Soft soil thickness/m	≤ 5	0.032	1
			5–10		2
			10–20		3
			20–30		4
			≥ 30		5
		Aquifer thickness/m	≤ 10	0.109	1
			10–20		2
			20–40		3
			40–60		4
			≥ 60		5
Land subsidence status	0.4	Rate of Land subsidence/mm a^−1^	≤ 10	0.266	1
			10–20		2
			20–30		3
			30–50		4
			≥ 50		5
		Cumulative surface subsidence volume/mm	≤ 10	0.133	1
			20–30		2
			30–50		3
			50–80		4
			≥ 80		5
Groundwater extraction	0.4	Groundwater level drawdown contour lines/m	≤ 20	0.4	1
			20–30		2
			30–40		3
			40–50		4
			≥ 50		5

$$H=\sum_{i=1}^{n}(Ai\times Bi)$$

In the above equation, *H* represents the comprehensive index value of risk, *A*_*i*_ denotes the score of the evaluation factor, *B*_*i*_ signifies the weight of the evaluation factor, and *n* stands for the number of evaluation factors.

Vulnerability, in the context of land Subsidence, relates to the degree of susceptibility of individuals, society, and property to threats and damages caused by land Subsidence. Victims of such disasters exhibit varying resilience levels and degrees of loss. Precise data acquisition, particularly at the local district (county) level, can be challenging. Therefore, vulnerability assessment based on urban planning units is adopted for better results, allowing for a comprehensive representation of economic, social, and environmental values through indicators like plot ratio and land use types. The evaluation factors for the vulnerability of land subsidence are selected as density of population (DP, the greater the population density, the greater the loss caused by land subsidence), building density (BD, the denser the buildings, the greater the loss caused by land subsidence), road density (RD, the closer to road traffic, the greater the loss caused by land subsidence), and functional zoning (FZ, the more complex and important the function of the zoning). The greater the impact of land subsidence hazard), the calculation method and evaluation method are the same as the risk, and the weight of each index is reshown in Table 2.

**Table2:** Land subsidence vulnerability evaluation index system and assignment.

Primary index	weight	Index classification	Importance assignment
Population density	0.449	< 500	1
		500–1000	2
		1000–3000	3
		3000–10,000	4
		> 10,000	5
Building density	0.205	0–20	1
		20–40	2
		40–60	3
		60–80	4
		80–100	5
Road density	0.239	< 0.68	1
		0.68–0.98	2
		0.98–1.13	3
		1.13–1.45	4
		> 1.45	5
Functional zoning	0.105	Small	1
		Lesser	2
		Intermediate	3
		Larger	4
		Big	5

## Results and discussion

### Land subsidence risk assessment

**Figure 5:**
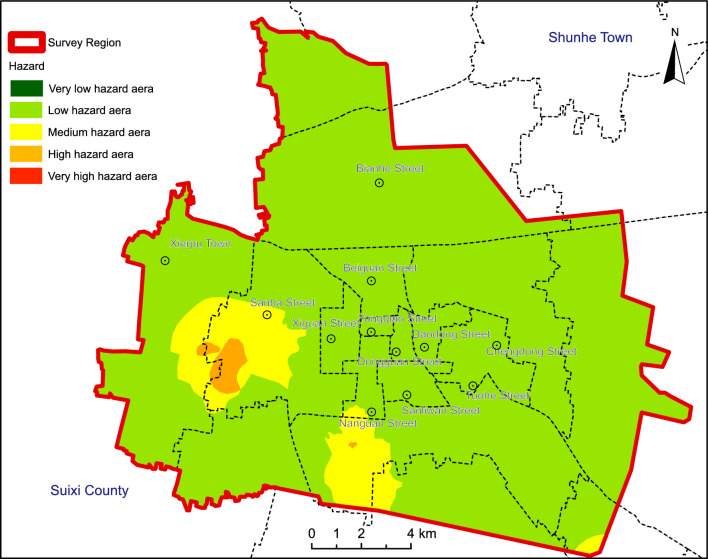
Hazard zoning diagram of land subsidenceof the Suzhou City. Arc GIS 10.8 platform (https://www.esri.com) was used for preparing the map for the study area.

Leveraging the spatial analysis capabilities of ArcGIS, a weighted overlay analysis of the land Subsidence hazard index was conducted to derive the hazard assessment results. As depicted in the land Subsidence hazard zoning map (Fig. 5), the following observations emerge: High-risk land Subsidence areas span approximately 2.65 km^2^, constituting 0.905% of the total area. Predominantly situated around Jiuli Village in the Sanba Street area and sporadic regions within the Xiecheng district, these areas fall within the concentrated groundwater extraction zone managed by Suzhou Water Supply Company. Moderate hazard areas encircle the high-risk zones, covering 23.09 km^2^ or 7.879% of the total area. These regions are primarily distributed around the periphery of Jiuli Village and Shili Village within the Sanba Street area, as well as in the vicinity of Dachen River in the Economic Development Zone and the groundwater extraction zone of the Xiecheng district. The majority of the study area, approximately 267.39 km^2^ or 91.214% of the total area, exhibits relatively low hazard levels.

**Figure 6:**
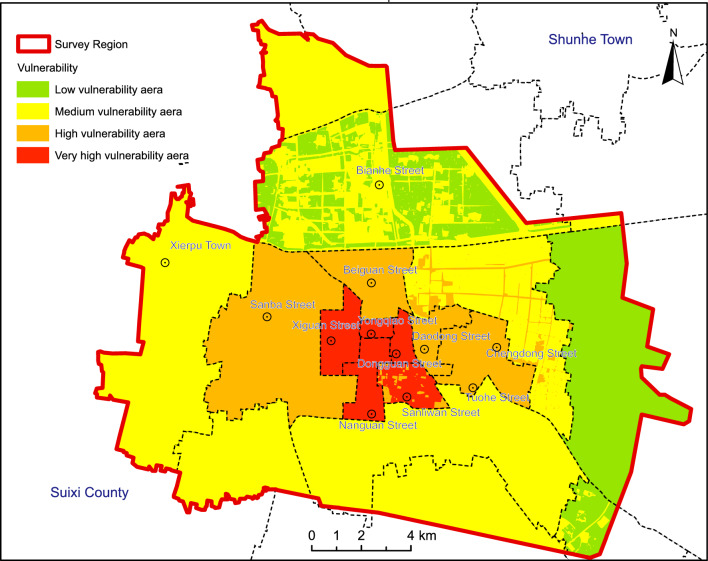
Vulnerability zoning diagram of land subsidence of the Suzhou City. Arc GIS 10.8 platform (https://www.esri.com) was used for preparing the map for the study area.

The vulnerability assessment results (Fig. 6) categorize the study area into high vulnerability, moderate-high vulnerability, moderate vulnerability, and low vulnerability zones: High vulnerability zones concentrate in West Gate Street, South Gate Street, East Gate Street, and Yongqiao Street, covering approximately 14.54 km^2^ or 4.955% of the total area. Areas with moderate-high vulnerability are mainly located in the Sanba Street, North Gate Street, Daodong Street, and Tuohe Street areas, as well as certain important main roads, encompassing about 52.87 km^2^ or 18.02% of the total area. Zones with moderate vulnerability are distributed in areas including Xierpu Town, the Economic Development Zone, City East Street, Zhuxianzhuang Town, Taoyuan Town, Daze Township, and Fuli Township, covering approximately 166.34 km^2^ or 56.68% of the total area. Regions with low vulnerability are concentrated in specific areas of Bianhe Street and Zhuxianzhuang Town, spanning around 59.71 km^2^ or 20.35% of the total area.

**Figure 7:**
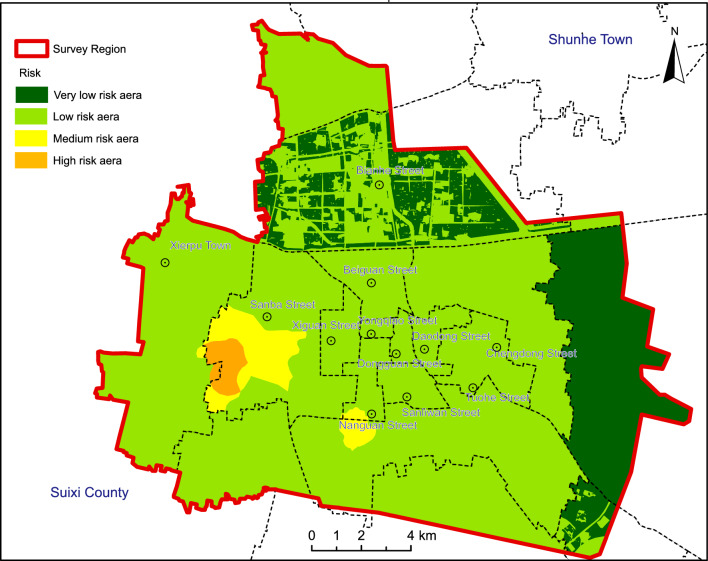
Risk zoning map of land subsidence of the Suzhou City. Arc GIS 10.8 platform (https://www.esri.com) was used for preparing the map for the study area.

Utilizing the spatial analysis capabilities of ARCGIS and adhering to the risk assessment model, a multiplication and overlay analysis of hazard and vulnerability was performed, resulting in the land Subsidence risk zoning map (Fig. 7). Noteworthy findings include: High-risk assessment areas align with high-vulnerability assessment areas, predominantly centered around Jiuli Village in the Sanba Street area, covering approximately 2.82 km^2^ or 0.96% of the total area. Moderate-risk assessment areas encircle the periphery of high-risk zones and are mainly concentrated around Jiuli Village, Shili Village, Dachen River in the Economic Development Zone, and the Xiecheng district, totaling about 9.18 km^2^ or 3.13% of the total area. Low-risk assessment areas correspond to the majority of the study area, encompassing about 222.24 km^2^ or 75.82% of the total area. The low-risk assessment zones are predominantly found in areas of Bianhe Street and Zhuxianzhuang Town, covering roughly 58.88 km^2^ or 20.09% of the total area.

### Suggestions for prevention and control of land subsidence risk

In recent years, Suzhou City has acknowledged the escalating importance of land Subsidence resulting from groundwater extraction. Over the period from 2016 to 2021, the city has proactively decommissioned more than 515 self-owned wells. In 2022, Suzhou City introduced the “Suzhou Underground Water Pressure Mining Replacement Plan.” This plan aims to decommission a total of 202 groundwater extraction wells in the over-mined zones of the Yongqiao District by 2025. This initiative is aligned with projects such as the Huaihe River North Diversion, Jiangji-Huai Phase II, and urban–rural water supply integration, reflecting Suzhou City's commitment to addressing land subsidence issues. The city has established a comprehensive monitoring infrastructure, including optical fiber monitoring holes, GPS monitoring points, and groundwater monitoring wells. Additionally, a 74 km leveling route across the urban area and continuous InSAR monitoring from 2019 to 2022 have provided crucial technical support for proactive land subsidence management.

**Figure 8:**
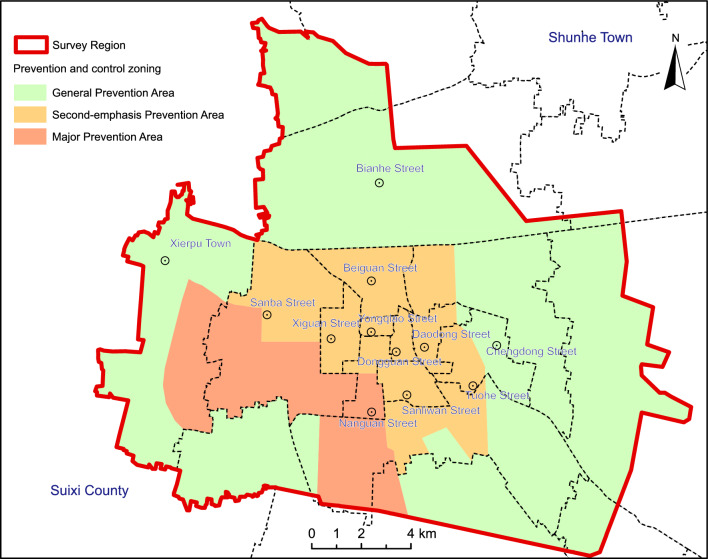
Prevention and control zoning of land subsidence of the Suzhou City. Arc GIS 10.8 platform (https://www.esri.com) was used for preparing the map for the study area.

In accordance with the outcomes of the land Subsidence risk assessment and in alignment for the Yongqiao District, the study area has been systematically divided into distinct zones to facilitate focused and effective prevention and control measures (Fig. 8). Specifically: Key Prevention and Control Zone for Water Source in the Western Urban Area and Economic Development Zone (41.21 km^2^–13.79% of the study area): This zone includes areas where land Subsidence has already occurred, primarily around Jiuli Village and the Suzhou Economic and Technological Development Zone. It encompasses a mix of urban and rural development, characterized by low-rise housing and significant industrial infrastructure. The zone has experienced early instances of land Subsidence, particularly in areas with concentrated groundwater extraction. Secondary Prevention and Control Zone for Yongqiao Central Urban Area (52.73 km^2^—17.99% of the study area): This zone covers areas where land Subsidence has not yet been observed, situated to the east of the Key Prevention and Control Zone. It occupies a central position within the urban core of the Yongqiao District, featuring high population density, diverse building types, major developmental projects, and robust transportation infrastructure. Due to substantial structural loads, this area is considered sensitive to potential land Subsidence. General Prevention and Control Zones (199.92 km^2^–68.22% of the study area): The remaining portions of the study area are designated as General Prevention and Control Zones, representing the majority of the study area. These zones do not exhibit significant land Subsidence risks.

Considering the ongoing trajectory of land subsidence development and concurrent urban planning initiatives in the Yongqiao area, coupled with the outcomes of the land subsidence risk zoning, the following recommendations are proposed for the prevention and control of land Subsidence:

(1) Integration of Land Subsidence Risk Assessments into Urban Planning and Development Strategies: It is recommended to incorporate comprehensive land subsidence risk assessments into urban planning and development strategies to preemptively address potential impacts in high-risk areas. Zoning regulations should be devised to restrict or regulate development in vulnerable regions, while promoting land use practices that mitigate subsidence risk. (2) Development of Construction Standards and Guidelines: Construction standards and guidelines should be formulated to account for land subsidence risks, ensuring the resilience and adaptability of new infrastructure. Encouragement of innovative building techniques and materials capable of withstanding or accommodating ground movements is advisable. (3) Reconfiguration of Water Resource Allocation: Within the subsidence-affected region, water resource allocation should be reconfigured by adjusting extraction horizons, timings, and mitigating excessive spatial and temporal concentration of extraction. Stringent controls on groundwater extraction volume are necessary to mitigate groundwater level depressions and reduce land subsidence. It is recommended to cease granting new groundwater extraction permits within restricted extraction areas, and to gradually reduce groundwater extraction. Additionally, addressing land subsidence challenges involves integrating surface water sources through initiatives such as river diversion projects. Promotion of alternative water sources, including rainwater harvesting and recycled wastewater, can diminish reliance on groundwater. (4) Policies for Natural Feature Protection and Restoration: Policies should be enacted to safeguard and rehabilitate natural features like wetlands and forests, which play a crucial role in stabilizing soil and mitigating subsidence risk. Implementation of environmental regulations to curtail activities contributing to land degradation and subsidence, such as excessive extraction and deforestation, is warranted. (5) Implementation of Regular and Systematic Monitoring: Land subsidence monitoring should be conducted through continuous leveling surveys, InSAR monitoring, stratified scale assessments, and groundwater monitoring. This approach furnishes dynamic data for analyzing present conditions, trends, and forecasting potentialities of land subsidence. Maintenance and management of land subsidence monitoring infrastructure should be sustained, with facilities retired and replenished based on operational realities to progressively refine the monitoring network. These recommendations collectively provide a holistic approach for policymakers, urban planners, and other stakeholders to create safer, more sustainable communities.

### Broader implications

Climate change has the potential to significantly alter precipitation patterns in the Yongqiao area, subsequently influencing the availability and distribution of water resources. Enhanced precipitation can trigger flood events, whereas decreased precipitation can lead to droughts, both of which exert considerable pressure on aquatic and terrestrial ecosystems^54,55^. Furthermore, changes in precipitation also affect groundwater in the area, which affects sedimentation. Therefore, the impact of climate change should also be taken into account in the future detection and prediction of land subsidence in this area.

A comprehensive assessment of the direct and indirect consequences of land subsidence on the local community, economy, and infrastructure is essential for a thorough comprehension of the issue and for prioritizing mitigation and adaptation strategies^56^. This phenomenon can adversely affect local businesses and agricultural activities reliant on stable ground conditions. Furthermore, land subsidence jeopardizes the quality and availability of groundwater, posing potential health risks associated with compromised drinking water sources. Additionally, subsidence-induced damage to roads, railways, and other transportation networks escalates maintenance expenses and disrupts transportation services. By quantifying the socio-economic impacts of land subsidence, policymakers and stakeholders can better grasp the urgency of the situation and make informed decisions regarding resource allocation and strategic planning for risk reduction and adaptation. Such information serves as a potent tool for conveying the importance of addressing land subsidence to the public and advocating for necessary actions. The scalability and adaptability of the risk assessment model utilized in the Yongqiao area of Suzhou City are pivotal for its broader application. To evaluate the model’s suitability for regions facing similar land subsidence issues, several considerations merit exploration. Firstly, modifications to accommodate diverse datasets, including the incorporation of proxy data when direct measurements are scarce, warrant attention. Secondly, emphasizing the modular nature of the model allows for the customization of risk indices tailored to regional characteristics. Thirdly, customization to different geographical contexts, encompassing varying geological formations, climatic conditions, and land use patterns, is paramount. Additionally, addressing uncertainty associated with the model’s predictions and providing guidelines for quantifying and communicating this uncertainty to stakeholders is imperative^57^. Lastly, elucidating how the model can inform policy development and planning across various scales, from local to national levels, enhances its practical utility^58^.

## Conclusions

In conclusion, this study leverages the ARCGIS platform and employs the Analytic Hierarchy Process (AHP) in conjunction with the comprehensive index method to conduct a meticulous analysis of diverse factors influencing subsidence in the research area. These factors include subsidence rate, cumulative settlement amount, groundwater level decline funnel, thickness of loose sediment layer, soft soil layer thickness, quantity of groundwater extraction layers, population density, building density, road traffic, and functional zoning. Through the integration of these factors into a comprehensive risk assessment model, the study area is classified into four levels: high, medium, low, and very low. The higher-risk region covers approximately 2.82 km^2^, constituting 0.96% of the total area, with a notable concentration in Jiuli Village within the Sanba Street locality.

Furthermore, an assessment of the current ground settlement conditions within the research area for the Yongqiao district, has led to the delineation of distinct intervention zones. The western urban source area is identified as the key control area, the downtown area as a sub-key control area, and the remaining expanse as the general control area. This stratification provides the basis for tailored control and management strategies. The recommendations include the judicious allocation of water resources, stringent oversight of groundwater extraction, implementation of innovative water conservation projects, and reinforcement of monitoring protocols. These proposals establish a decisive framework for upcoming initiatives focused on averting and alleviating land subsidence disasters, guiding urban planning and construction endeavors, and promoting sustainable development.

### Supplementary Information

Supplementary Information.

## Data Availability

All data generated or analysed during this study are included in this published article [and its supplementary information files].
